# Cytotoxicity and activation of the Wnt/beta-catenin pathway in mouse embryonic stem cells treated with four GSK3 inhibitors

**DOI:** 10.1186/1756-0500-7-273

**Published:** 2014-04-29

**Authors:** Ortwin Naujok, Jana Lentes, Ulf Diekmann, Claudia Davenport, Sigurd Lenzen

**Affiliations:** 1 Institute of Clinical Biochemistry, Hannover Medical School, Carl-Neuberg-Str. 1, Hannover 30625, Germany

## Abstract

**Background:**

Small membrane-permeable molecules are now widely used during maintenance and differentiation of embryonic stem cells of different species. In particular the glycogen synthase kinase 3 (GSK3) is an interesting target, since its chemical inhibition activates the Wnt/beta-catenin pathway. In the present comparative study four GSK3 inhibitors were characterized.

**Methods:**

Cytotoxicity and potential to activate the Wnt/beta-catenin pathway were tested using the commonly used GSK3 inhibitors BIO, SB-216763, CHIR-99021, and CHIR-98014. Wnt/beta-catenin-dependent target genes were measured by quantitative PCR to confirm the Wnt-reporter assay and finally EC_50_-values were calculated.

**Results:**

CHIR-99021 and SB-216763 had the lowest toxicities in mouse embryonic stem cells and CHIR-98014 and BIO the highest toxicities. Only CHIR-99021 and CHIR-98014 lead to a strong induction of the Wnt/beta-catenin pathway, whereas BIO and SB-216763 showed a minor or no increase in activation of the Wnt/beta-catenin pathway over the natural ligand Wnt3a. The data from the Wnt-reporter assay were confirmed by gene expression analysis of the TCF/LEF regulated gene *T*.

**Conclusions:**

Out of the four tested GSK3 inhibitors, only CHIR-99021 and CHIR-98014 proved to be potent pharmacological activators of the Wnt/beta-catenin signaling pathway. But only in the case of CHIR-99021 high potency was combined with very low toxicity.

## Background

Small molecules are attractive chemical compounds to control pluripotency in murine embryonic stem cells [1-6]. They may also be used to direct embryonic stem (ES) cell fates during differentiation [7-10] or enhance the reprogramming of adult cell types into iPS cells [11]. In contrast to growth factors or cytokines, they offer distinct advantages such as a well-defined activity, good stability upon heat exposure in a cell culture incubator, less batch-to-batch differences, and modest costs. Chemical inhibitors of the glycogen synthase kinase 3 (GSK3) are very attractive tools as they allow the control of pluripotency in mouse and rat ES cells [5,6] and may be used to direct human ES cells into mesodermal or endodermal cell fates via activation of the canonical Wnt-pathway [12-16].

The Wnt/beta-catenin pathway controls miscellaneous biological processes during tissue development by autocrine and paracrine activities [17]. When Wnt-signaling is activated by binding of secreted Wnt-protein to its receptor, dishevelled (Dvl/Dsh) is recruited and inhibits the GSK3 located in the beta-catenin destruction complex [18]. This leads to an accumulation of free non-phosphorylated beta-catenin in the cytosol, which translocates to the nucleus and transactivates Wnt-target genes together with the T-cell factor (TCF)/lymphoid-enhancing factor (LEF) family of transcription factors [18]. Thus, chemical inhibition of the GSK3 (herewith referred to as GSK3i) leads to a pharmacological activation of the canonical Wnt-signaling pathway. However, the compound to be used in a study should be carefully selected as small molecules may exhibit cytotoxicity, side effects, and differ in activity.

In this study the effect of four commonly used GSK3 inhibitors, namely BIO, SB-216763, CHIR-99021, and CHIR-98014 was analyzed in a comparative fashion in two different mouse embryonic stem cell lines (ES-D3 and ES-CCE). Specifically, the cytotoxicity, the ability to activate the Wnt/beta-catenin-pathway, and the changes in gene and protein expression were analyzed in a defined serum-reduced medium devoid of LIF.

The results show that one of the four tested compounds, CHIR-99021, is optimally suited for strong activation of the Wnt/beta-catenin-pathway without significant concomitant toxicity.

## Methods

### Materials

RPMI1640 advanced, KO-DMEM, NEAA, and glutamax were obtained from Life Technologies (Darmstadt, Germany) and fetal calf serum was from (PAA Laboratories/GE Healthcare, Cölbe, Germany). The GSK3 inhibitors BIO, SB-216763, and CHIR-99021, were from Tocris Bioscience (Wiesbaden-Nordenstadt, Germany) and CHIR-98014 was purchased from Axon Medchem (Groningen, Netherlands). Wnt3a was obtained from Peprotech (Hamburg, Germany) and Matrigel was from Corning (Corning, NY, USA). All primers were synthesized by Life Technologies. The RevertAid™ H-Minus M-MuLV reverse transcriptase was purchased from Thermo Fisher Scientific (Braunschweig, Germany). The GoTaq® Taq polymerase was from Promega (Mannheim, Germany) and dNTPs from Genecraft (Münster, Germany). Unless mentioned otherwise, chemicals were obtained from Sigma-Aldrich (Taufkirchen, Germany).

### Mouse ES cell culture

To maintain the pluripotency of the mouse ES cell lines ES-D3 [19] and ES-CCE [20], they were routinely cultured on gelatine-coated dishes in KO-DMEM containing 25 mM glucose supplemented with 15% FCS, 2 mM L-glutamine, 100 μM NEAA, 100 μM 2-mercaptoethanol, penicillin/streptomycin, and 1,000 U/ml LIF (eBiosciences, Frankfurt, Germany) [10,21]. The medium was changed daily and the cells were passaged 2–3 times per week. The basal medium for the comparative experiments was RPMI advanced supplemented with 0.2% FCS, penicillin/streptomycin and 1-fold glutamax with different concentrations of BIO, SB-216763, CHIR-99021 and CHIR-98014. A randomized control was performed with basal medium without growth factors and/or small molecules. Physiological activation of the Wnt/beta-catenin-pathway was tested in medium supplemented with 50 ng/ml Wnt3a.

### Cell viability assay

The viability of the mouse ES cells was determined after exposure to different concentrations of GSK3 inhibitors for three days using the MTT assay [22]. The decrease of MTT activity is a reliable metabolism-based test for quantifying cell viability; this decrease correlates with the loss of cell viability. 2,000 cells were seeded overnight on gelatine-coated 96-well plates in LIF-containing ES cell medium. On the next day the medium was changed to medium devoid of LIF and with reduced serum and supplemented with 0.1 – 1 μM BIO, or 1 – 10 μM SB-216763, CHIR-99021 or CHIR-98014. Basal medium without GSK3 inhibitors or DMSO was used as control. All tested conditions were analyzed in triplicates.

### Wnt/beta-catenin activity assay

The Wnt/beta-catenin reporter assay was performed with the M50 Super 8× TOPFlash and M51 Super 8× FOPFlash vector containing the firefly luciferase gene under the control of TCF/LEF binding sites (M50 Super 8x TOPFlash) or mutated bindings sites (M51 Super 8× FOPFlash) [23]. 12,500 cells were seeded overnight on gelatine-coated 96-well plates in LIF-containing ES cell medium. On the next day the cells were transfected using Lipofectamine (Life Technologies) with one of the aforementioned vectors plus pGL4.75 [hRluc/CMV] (Promega) encoding the renilla luciferase reporter gene hRluc as a transfection control. Six hours after transfection the medium was changed to medium devoid of LIF, with reduced serum, and supplemented with 0.5 μM Bio, 5 μM SB-2167763, 5 μM CHIR-99021 and 1 μM CHIR-98014. The Dual-Luciferase® reporter assay system (Promega) was employed 48 and 72 h after medium change to follow the luminescence reaction using a GloMax®-multi detection system (Promega).

### Gene expression analyses

Total RNA was isolated from the cells using the RNeasy Kit (Qiagen, Hilden, Germany). Briefly, the cells were lysed in Qiazol (Qiagen), the hydrophilic phase was loaded onto RNA spin columns, and RNA was then prepared as instructed. cDNA synthesis was performed with random hexamer primers and 2 μg of the isolated total RNA following the manufacturer’s instructions. 10–20 ng of cDNA was then loaded in each well of a 384-well plate and specific primers were mixed with the GoTaq® PCR master mix according to the manufacturers’ instructions. Primer sequences were (5’-3’): *T* fw: catcggaacagctctccaacctat, rev: gtgggctggcgttatgactca, *Nanog* fw: ccctgaggaggaggagaacaaggtc, rev: ccactggtttttctgccaccgc, and *Pou5f1* fw: aggcccggaagagaaagcgaacta, rev: tgggggcagaggaaaggatacagc. Each qPCR amplification was performed in triplicates and the gathered data were normalized with qBasePlus (Biogazelle, Zwijnaarde, Belgium) against the housekeeping genes *G6pdx*, *Tbp*, and *Tuba1a*[10].

### Immunofluorescence staining

Immunofluorescence was performed according to standard procedures. ES cells were treated on 6-well plates with different GSK3 inhibitors and after 48 h 100,000 cells were re-seeded in each cavity of Matrigel-coated glass slides (Zellkontakt, Nörten-Hardenberg, Germany). After 24 h in medium the cells were fixed in 4% (w/v) paraformaldehyde for 45 min at 4°C. Subsequently, the cells were blocked for 20 min in PBS plus 0.2% Triton X-100, 6% BSA, and 1 mg/ml NaBH_4_. Primary and secondary antibodies were diluted in PBS with 0.1% Triton X-100 and 0.1% BSA. Primary antibodies were incubated on the slides for 2.5 h at room temperature (RT) or overnight at 4°C. Secondary antibodies were incubated on the slides for 1 h at RT. The following primary antibodies were used: anti-Oct3/4 (sc-5279, Santa Cruz Biotechnology, Heidelberg, Germany) and anti-Brachyury (AF2085, R&D Systems, Minneapolis, MN, USA). Secondary antibodies were obtained from Dianova (Hamburg, Germany). Finally, the slides were mounted with immunoselect antifading mounting medium containing DAPI (Dianova) to counterstain the nuclei. The stained cells were examined using an Olympus IX81 inverted microscope (Olympus, Hamburg, Germany).

### Statistics

Data were expressed as mean values ± SEM unless stated otherwise. Statistical analyses were performed using the GraphPad Prism software (Graphpad, San Diego, CA, USA) applying *Student’s t-test* or ANOVA followed by *Bonferroni’s* or *Dunnett’s post hoc* test for multiple comparisons.

## Results

### Cell viability

**Figure 1:**
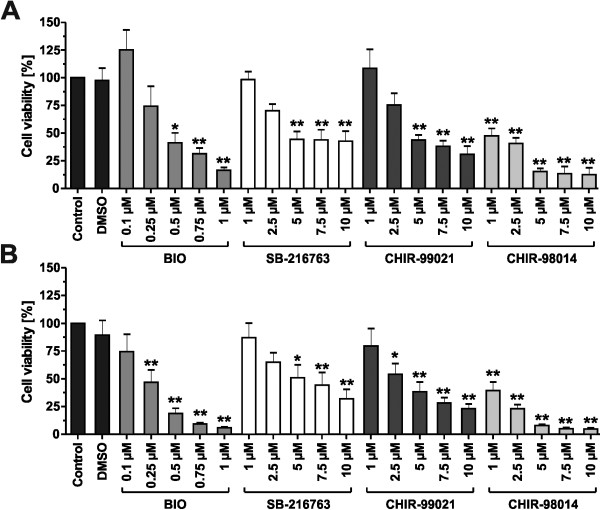
**Cell viability of mouse embryonic stem cells after exposure to GSK3 inhibitors. (A)** Cell viability of the mouse embryonic stem cell line ES-D3 after a three day treatment with different concentrations of BIO, SB-216763, CHIR-99021 and CHIR-98014. Cell viability was measured with the MTT assay. DMSO without inhibitors was used as vehicle control. Data are expressed as percentages normalized to the control condition without inhibitors. **(B)** Cell viability of the mouse embryonic stem cell line ES-CCE after a three day treatment with different concentrations of BIO, SB-216763, CHIR-99021 and CHIR-98014. Values are means ± SEM, n = 3-5. ANOVA plus *Dunnett’s post hoc* test. ** p ≤ 0.01, * p ≤ 0.05 compared to the control.

Two mouse stem cell lines, ES-D3 and ES-CCE, were exposed to different concentrations of the GSK3 inhibitors BIO, SB-216763, CHIR-99021 and CHIR-98014 in defined medium devoid of LIF and with low FCS. Their toxicity was tested using the MTT assay; Bio at concentrations of 0.1 to 1 μM and all other compounds at 1 – 10 μM. As controls the cells were treated without compounds in the medium with the solvent DMSO as vehicle control. Differentiation in the presence of DMSO did not significantly decrease cell viability in both mouse ES cell lines (Figure 1A/B). However, toxic effects of the GSK3 inhibitors were observed for all compounds.

In detail the viability of ES-D3 cells was reduced by 25.7% at 0.25 μM, 58.7% at 0.5 μM, 68.7% at 0.75 μM and 83.5% at 1 μM BIO. Calculation of the half maximal inhibitory concentration yielded in an IC_50_ of 0.48 μM for BIO. In presence of SB-216763 the viability of the ES-D3 cells was reduced by 1.7% at 1 μM, 29.8% at 2.5 μM, 55.6% at 5 μM, 56.1% at 7.5 μM and 57.2% at 10 μM SB-216763 with an IC_50_ of 5.7 μM. In the presence of CHIR-99021 the viability of the ES-D3 cells was reduced by 24.7% at 2.5 μM, 56.3% at 5 μM, 61.9% at 7.5 μM and 69.2% at 10 μM CHIR-99021 with an IC_50_ of 4.9 μM. CHIR-98014 reduced the viability by 52% at 1 μM and showed the greatest toxicity with increasing concentrations. The IC_50_ of CHIR-98014 was 1.1 μM (Figure 1A). ES-CCE cells generally showed a higher toxicity after GSK3i exposure (Figure 1B).

### Wnt/beta-catenin pathway activation

**Figure 2:**
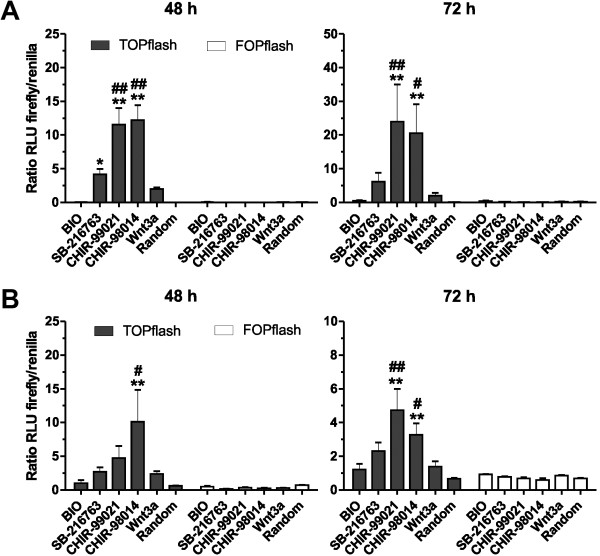
**Activation of the canonical Wnt-pathway by GSK3 inhibition. (A, B)** Assessment of the Wnt/beta-catenin pathway activity after exposure to the GSK3 inhibitors BIO (0.5 μM), SB-216763 (5 μM), CHIR-99021 (5 μM) or CHIR-98014 (1 μM) in **(A)** ES-D3 cells and **(B)** ES-CCE cells using the M50 Super 8x TOPflash luminescence reporter system. A M51 Super 8x FOPflash vector with mutated TCF/LEF binding sites was used as a negative control. As a positive control, the ES cells were incubated with 50 ng/ml Wnt3a. Values are means of the relative light units of the firefly and renilla luciferase luminescence ratio ± SEM, n = 6. ** p < 0.01, * p < 0.05 compared to randomly treated cells, ## < 0.01, # < 0.05 Wnt3a treated cells, ANOVA with *Dunnett’s post hoc* test.

To assess the activation of the Wnt/beta-catenin signaling pathway by GSK3i, a dual luciferase reporter assay was used (Figure 2A/B). Mouse ES cells were transfected with the M50 TOPflash firefly luciferase vector, comprising seven TCF/LEF binding sites or as a negative control with the M51 FOPflash vector harboring mutated binding sites [23]. To normalize the transfection efficiency, a hRluc/CMV vector with a constitutive expression of the renilla luciferase was used. Mouse ES cells of both lines were incubated with GSK3 inhibitors with concentrations close to the IC_50_ values calculated for the ES-D3 cell line. Dual luciferase luminescence was measured 48 and 72 h after cultivation with one of the inhibitors. In ES-D3 cells (Figure 2A) cultivation with CHIR-99021 and CHIR-98014 resulted in a significant activation of the Wnt/beta-catenin pathway. The measured luminescence signals were significantly increased compared to control cells without inhibitors and to cells, which were incubated with 50 ng/ml Wnt3a. In the presence of SB-216763, the luminescence signal was also significantly increased compared to control cells but not to Wnt3a-incubated cells. This pattern was nearly identical for the 72 h dataset. Incubation with BIO did not result in a robust activation of the Wnt/beta-catenin pathway above controls (Figure 2A).

A similar pattern with slight differences was detected for ES-CCE cells (Figure 2B). Treatment of ES-CCE cells with CHIR-98014 showed a significant increase of Wnt-signaling after 48 and 72 h, whereas CHIR-99021 showed only a significant increase at 72 h compared to random controls or Wnt3a-treated cells. In contrast to ES-D3 cells, a higher luminescence noise was detected for the M51 FOPflash vector transfections, which increased with time. Incubation with BIO or SB-216763 resulted in detectable luminescence signals, which were nevertheless comparable to that of negative or positive controls (Figure 2B).

### Changes in gene expression during differentiation and incubation with GSK3 inhibitors

**Figure 3:**
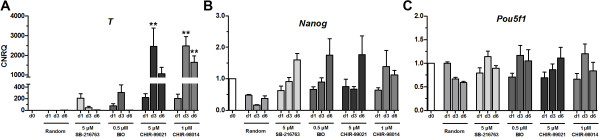
**Gene expression changes upon treatment with GSK3 inhibitors. (A-C)** qPCR analysis of *T* and the pluripotency markers *Nanog* and *Pou5F1* after a six day treatment of mouse ES-D3 cells with BIO (0.5 μM), SB-216763 (5 μM), CHIR-99021 (5 μM), or CHIR-98014 (1 μM). Randomized differentiation without any growth factors or small molecules was used as a control. Values are calibrated normalized relative quantities (CNRQ) after normalization to three stably expressed housekeeping genes and scaling to undifferentiated ES cells. Values are means ± SEM, n = 4–5. ** p < 0.01, * p < 0.05 compared to randomly differentiated ES cells of the same day, ANOVA with *Dunnett’s post hoc* test.

A gene expression profile of the Wnt-regulated gene *T* and the pluripotency master regulators *Pou5F1* and *Nanog* was measured in ES-D3 cells after six days of treatment with 0.5 μM BIO, 5 μM SB-216763, 5 μM CHIR-99021, and 1 μM CHIR-98014 (Figure 3). As a negative control, the cells were incubated without inhibitors or growth factors (random). A significant effect was observed for the *T* gene expression, which was induced up to 2,500-fold in CHIR-99021 or CHIR-98014 treated cells (Figure 3A). The induction by BIO and SB-216763 was lower but still up to 300-fold higher compared to undifferentiated or randomly differentiated cells. Maximal induction of the *T* gene expression in the presence of GSKi was detected after three days of differentiation (Figure 3A). The gene expression of *Nanog* decreased in randomly treated control cells, whereas in samples cultivated with GSK3i it showed an increased expression (Figure 3B). Comparably, *Pou5F1* gene expression decreased over time in random controls whereas GSK3i did not significantly change the gene expression (Figure 3C).

### Immunofluorescence staining of Brachyury and Oct3/4 in ES-D3 cells treated with GSK3 inhibitors

**Figure 4:**
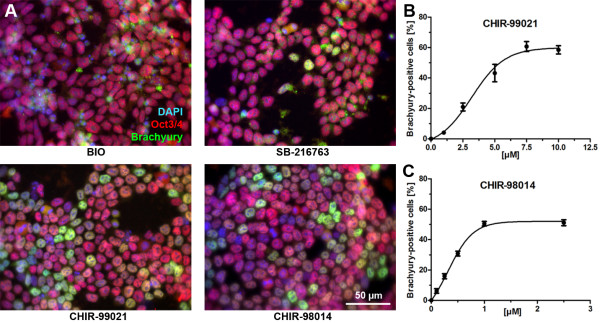
**Immunofluorescence staining of Brachyury and Oct3/4 in ES-D3 cells treated with GSK3 inhibitors. (A)** Fluorescence micrographs showing the protein expression of Brachyury/Oct3/4 after a three day differentiation of ES-D3 cells with BIO (0.5 μM), SB-216763 (5 μM), CHIR-99021 (5 μM), or CHIR-98014 (1 μM). Nuclei were counterstained with DAPI (blue). The merge is shown in yellow. Original magnification 400x. Scale bar = 50 μm. Quantitative analysis of Brachyury-positive cells after a three day treatment with of 0 – 10 μM CHIR-99021 **(B)** and 0 – 2.5 μM CHIR-98014 **(C)**. Values are means ± SEM of six analyzed images of 2 different experiments.

The *T* gene has been reported as a TCF/beta-catenin target gene [24]. Thus, immunostaining of Brachyury and counter-staining for Oct3/4 was performed to verify that GSK3i was functionally relevant for the Wnt/beta-catenin signaling pathway in mouse ES cells. Immunofluorescence staining of Brachyury and Oct3/4 showed almost no Brachyury-positive cells after a three day treatment with 0.5 μM BIO (Figure 4A). In contrast, almost all cells were positive for Oct3/4. After treatment with SB-216763, all cells were positive for Oct3/4 and ~3% were double-positive for Brachyury and Oct3/4. Both transcription factors were localized at the nucleus and during cell division the fluorescence signal was observed in the cytoplasm. Treatment with CHIR-99021 (5 μM) or CHIR-98014 (1 μM) yielded around 43% or 50% Brachyury-positive cells, respectively, from which the majority was additionally positive for Oct3/4 (Figure 4A). Counting of cells positively stained for Brachyury revealed a concentration-response curve for CHIR-99021 and CHIR-98014 with saturation of the curve at 60% or 50% Brachyury-positive cells, respectively (Figure 4B/C). EC_50-_values were 3.19 μM for CHIR-99021 and 0.32 μM for CHIR-98014.

## Discussion

In this comparative study the effect of four commonly used GSK3 inhibitors on mouse ES cells was studied with respect to their cytotoxicity and the potential to activate the canonical Wnt-signaling pathway.

The two studied mouse ES cell lines originate from the same mouse strain and were isolated using the same methods [19,20], nevertheless they responded differently to a treatment with GSK3 inhibitors. The ES-CCE cell line was more prone to GSK3i induced cell death then the ES-D3 cell line. In addition, the activity of these compounds was different in the two cell lines. SB-216763 and CHIR-99021 caused the smallest reduction of the viability in both lines, whereas CHIR-98014 induced a high rate of cell death in ES-D3 cells and in particular in ES-CCE cells. The small molecule BIO, which has been used in a number of embryonic stem cell studies [25-27], was strongly cytotoxic in both ES cell lines already at concentrations below 1 μM. This is a surprising result, since other studies used this inhibitor at much higher concentrations (2-5 μM) [25-28]. In these studies the cytotoxicity was not tested and the inhibitor was used in presence of a high fetal calf serum concentration or knock-out serum replacement concentration, which might have quenched the cytotoxic effect.

With respect to the effect on the canonical Wnt-pathway, CHIR-99021 and CHIR-98014 showed the greatest activation potential, which was significantly higher than that of Wnt3a. Thus, GSK3i by these two compounds is a more potent chemical hyperactivator of the canonical Wnt-pathway than the natural ligand Wnt3a.

Surprisingly, SB-216763 caused a weaker activation of the Wnt/beta-catenin-pathway in the TOPflash-assay comparable to that of Wnt3a. This proves that this compound is less suited for GSK3i than the two CHIR-inhibitors. An activation of the Wnt-pathway by BIO in ES-D3 was barely detectable. Only in ES-CCE cells a distinct signal, delayed by 24 h, could be detected. Higher concentrations could not be analyzed due to the high cytotoxicity. Bain and co-workers recommended CHIR-99021 as the most selective GSK3 inhibitor although the tests in this study were performed in a cell-free system and potential cytotoxic effects were not analyzed [29]. Next to the Wnt/beta-catenin-pathway the GSK3 fulfills a myriad of cellular functions [18]. Thus, some effects of the treatment with GSK3 inhibitors might not be related to the Wnt-pathway alone. In this line it is even more important to verify a robust activation of the Wnt-pathway either by analyzing its activity or analyzing proper targets downstream of Wnt/beta-catenin.

It has been previously reported that double-knockout of both GSK3 isoforms in mouse ES cells yielded an induction of *T*, *Pou5F1* (Oct3/4) and *Nanog* upon differentiation [30]. In the present study we analyzed these three genes during culture conditions that would allow randomized differentiation of mouse ES cells. In confirmation to previous results [30], gene expression of both pluripotency markers was not decreased in the presence of GSK3i, whereas it was decreased in random controls. This is in line with earlier studies reporting the maintenance of expression of pluripotency factors by GSK3i [3,4,31]. Several studies reported that the inhibition of the GSK3, FGF- and ERK-signaling pathways resulted in improved derivation of new mouse ES cell lines [32,33]. These findings lead to the development of the 3i and 2i media, which are now routinely used for the derivation and culture of mouse and rat ES cells [5,6].

Additionally, a strong increase in gene expression of *T* was observed upon GSK3i. The strongest effect was detected for CHIR-99021 and CHIR-98014, which confirms the reporter assay results. The increased gene expression yielded Brachyury-positive cells predominantly together with Oct3/4 in case of both CHIRs but not in the presence of BIO and SB-216763. Interestingly, only a small subpopulation accounted for the 2,500-fold increase in *T* expression as measured by qPCR. This observed heterogeneity raises the question why some cells did not acquire Brachyury-positivity whereas another subpopulation showed a significantly increased Brachyury expression.

## Conclusions

Small molecule inhibitors of GSK3 have become valuable reagents for studies on pluripotent stem cells. In murine ES cells they may be used to maintain pluripotency whereas in human ES cells GSKi was shown to be useful in differentiation experiments [15,25]. The results presented in this study show that mouse ES cell lines can respond differently to GSK3i. Thus, effective and non-toxic concentrations of GSK3 inhibitors and the duration of their treatment need to be carefully titrated for each mouse ES cell line. Two strong GSK3 inhibitors, namely CHIR-99021 and CHIR-98014, were identified and characterized. These inhibitors allowed a pharmacological hyperactivation of the Wnt/beta-catenin signaling pathway in mouse ES cells, more potently than that achieved by the natural ligand Wnt3a. Thus, these GSK3 inhibitors are very useful compounds for further experimentation with pluripotent cells.

## Competing interests

The authors declare that they have no competing interests.

## Authors’ contributions

ON designed the study, analyzed and interpreted the data and wrote the manuscript. JL collected and interpreted the cytotoxicity and reporter assay data. UD analyzed and interpreted the data and wrote the manuscript. CD performed immunofluorescence and real-time PCR and wrote the manuscript. SL designed the study, wrote and finally approved the manuscript. All authors read and approved the final manuscript.

## References

[B1] Bone HK Damiano T Bartlett S Perry A Letchford J Ripoll YS Nelson AS Welham MJ Involvement of GSK-3 in regulation of murine embryonic stem cell self-renewal revealed by a series of bisindolylmaleimides Chem Biol 2009 16 15 27 10.1016/j.chembiol.2008.11.003 19171302

[B2] Ye S Tan L Yang R Fang B Qu S Schulze EN Song H Ying Q Li P Pleiotropy of glycogen synthase kinase-3 inhibition by CHIR99021 promotes self-renewal of embryonic stem cells from refractory mouse strains PloS one 2012 7 e35892 10.1371/journal.pone.0035892 22540008 PMC3335080

[B3] Kirby LA Schott JT Noble BL Mendez DC Caseley PS Peterson SC Routledge TJ Patel NV Glycogen synthase kinase 3 (GSK3) inhibitor, SB-216763, promotes pluripotency in mouse embryonic stem cells PloS one 2012 7 e39329 10.1371/journal.pone.0039329 22745733 PMC3383737

[B4] Sanchez-Ripoll Y Bone HK Owen T Guedes AM Abranches E Kumpfmueller B Spriggs RV Henrique D Welham MJ Glycogen synthase kinase-3 inhibition enhances translation of pluripotency-associated transcription factors to contribute to maintenance of mouse embryonic stem cell self-renewal PloS one 2013 8 e60148 10.1371/journal.pone.0060148 23577087 PMC3618116

[B5] Li P Tong C Mehrian-Shai R Jia L Wu N Yan Y Maxson RE Schulze EN Song H Hsieh CL Pera MF Ying QL Germline competent embryonic stem cells derived from rat blastocysts Cell 2008 135 1299 1310 10.1016/j.cell.2008.12.006 19109898 PMC2735113

[B6] Nichols J Jones K Phillips JM Newland SA Roode M Mansfield W Smith A Cooke A Validated germline-competent embryonic stem cell lines from nonobese diabetic mice Nat Med 2009 15 814 818 10.1038/nm.1996 19491843

[B7] Chen S Borowiak M Fox JL Maehr R Osafune K Davidow L Lam K Peng LF Schreiber SL Rubin LL Melton D A small molecule that directs differentiation of human ESCs into the pancreatic lineage Nat Chem Biol 2009 5 258 265 10.1038/nchembio.154 19287398

[B8] Zhu S Wurdak H Wang J Lyssiotis CA Peters EC Cho CY Wu X Schultz PG A small molecule primes embryonic stem cells for differentiation Cell stem cell 2009 4 416 426 10.1016/j.stem.2009.04.001 19427291

[B9] Borowiak M Maehr R Chen S Chen AE Tang W Fox JL Schreiber SL Melton DA Small molecules efficiently direct endodermal differentiation of mouse and human embryonic stem cells Cell stem cell 2009 4 348 358 10.1016/j.stem.2009.01.014 19341624 PMC4564293

[B10] Naujok O Lenzen S A critical re-evaluation of CD24-positivity of human embryonic stem cells differentiated into pancreatic progenitors Stem Cell Rev 2012 8 779 791 10.1007/s12015-012-9362-y 22529013

[B11] Schmole AC Hubner R Beller M Rolfs A Frech MJ Small molecules in stem cell research Curr Pharm Biotechnol 2013 14 36 45 23092256

[B12] D'Amour KA Bang AG Eliazer S Kelly OG Agulnick AD Smart NG Moorman MA Kroon E Carpenter MK Baetge EE Production of pancreatic hormone-expressing endocrine cells from human embryonic stem cells Nat Biotechnol 2006 24 1392 1401 10.1038/nbt1259 17053790

[B13] Mfopou JK Chen B Mateizel I Sermon K Bouwens L Noggin, retinoids, and fibroblast growth factor regulate hepatic or pancreatic fate of human embryonic stem cells Gastroenterology 2010 138 2233 2245 10.1053/j.gastro.2010.02.056 20206178

[B14] Kroon E Martinson LA Kadoya K Bang AG Kelly OG Eliazer S Young H Richardson M Smart NG Cunningham J Agulnick AD D'Amour KA Carpenter MK Baetge EE Pancreatic endoderm derived from human embryonic stem cells generates glucose-responsive insulin-secreting cells in vivo Nat Biotechnol 2008 26 443 452 10.1038/nbt1393 18288110

[B15] Tan JY Sriram G Rufaihah AJ Neoh KG Cao T Efficient derivation of lateral plate and paraxial mesoderm subtypes from human embryonic stem cells through GSKi-mediated differentiation Stem Cells Dev 2013 22 1893 1906 10.1089/scd.2012.0590 23413973 PMC3685395

[B16] Sui L Bouwens L Mfopou JK Signaling pathways during maintenance and definitive endoderm differentiation of embryonic stem cells Int J Dev Biol 2013 57 1 12 10.1387/ijdb.120115ls 23585347

[B17] Holland JD Klaus A Garratt AN Birchmeier W Wnt signaling in stem and cancer stem cells Curr Opin Cell Biol 2013 25 254 264 10.1016/j.ceb.2013.01.004 23347562

[B18] Doble BW Woodgett JR GSK-3: tricks of the trade for a multi-tasking kinase J Cell Sci 2003 116 1175 1186 10.1242/jcs.00384 12615961 PMC3006448

[B19] Doetschman TC Eistetter H Katz M Schmidt W Kemler R The in vitro development of blastocyst-derived embryonic stem cell lines: formation of visceral yolk sac, blood islands and myocardium J Embryol Exp Morphol 1985 87 27 45 3897439

[B20] Robertson E Bradley A Kuehn M Evans M Germ-line transmission of genes introduced into cultured pluripotential cells by retroviral vector Nature 1986 323 445 448 10.1038/323445a0 3762693

[B21] Diekmann U Elsner M Fiedler J Thum T Lenzen S Naujok O MicroRNA target sites as genetic tools to enhance promoter-reporter specificity for the purification of pancreatic progenitor cells from differentiated embryonic stem cells Stem Cell Rev 2013 9 555 568 10.1007/s12015-012-9416-1 23111459

[B22] Naujok O Kaldrack J Taivankhuu T Jörns A Lenzen S Selective removal of undifferentiated embryonic stem cells from differentiation cultures through HSV1 thymidine kinase and ganciclovir treatment Stem Cell Rev 2010 6 450 461 10.1007/s12015-010-9148-z 20411442

[B23] Veeman MT Slusarski DC Kaykas A Louie SH Moon RT Zebrafish prickle, a modulator of noncanonical Wnt/Fz signaling, regulates gastrulation movements Curr Biol 2003 13 680 685 10.1016/S0960-9822(03)00240-9 12699626

[B24] Arnold SJ Stappert J Bauer A Kispert A Herrmann BG Kemler R Brachyury is a target gene of the Wnt/beta-catenin signaling pathway Mech Dev 2000 91 249 258 10.1016/S0925-4773(99)00309-3 10704849

[B25] Bone HK Nelson AS Goldring CE Tosh D Welham MJ A novel chemically directed route for the generation of definitive endoderm from human embryonic stem cells based on inhibition of GSK-3 J Cell Sci 2011 124 1992 2000 10.1242/jcs.081679 21610099 PMC3104033

[B26] Sato H Amagai K Shimizukawa R Tamai Y Stable generation of serum- and feeder-free embryonic stem cell-derived mice with full germline-competency by using a GSK3 specific inhibitor Genesis 2009 47 414 422 10.1002/dvg.20514 19391115 PMC2726955

[B27] Besser D Expression of nodal, lefty-a, and lefty-B in undifferentiated human embryonic stem cells requires activation of Smad2/3 J Biol Chem 2004 279 45076 45084 10.1074/jbc.M404979200 15308665

[B28] Sato N Meijer L Skaltsounis L Greengard P Brivanlou AH Maintenance of pluripotency in human and mouse embryonic stem cells through activation of Wnt signaling by a pharmacological GSK-3-specific inhibitor Nat Med 2004 10 55 63 10.1038/nm979 14702635

[B29] Bain J Plater L Elliott M Shpiro N Hastie CJ McLauchlan H Klevernic I Arthur JS Alessi DR Cohen P The selectivity of protein kinase inhibitors: a further update Biochem J 2007 408 297 315 10.1042/BJ20070797 17850214 PMC2267365

[B30] Doble BW Patel S Wood GA Kockeritz LK Woodgett JR Functional redundancy of GSK-3alpha and GSK-3beta in Wnt/beta-catenin signaling shown by using an allelic series of embryonic stem cell lines Dev Cell 2007 12 957 971 10.1016/j.devcel.2007.04.001 17543867 PMC4485918

[B31] Wray J Kalkan T Gomez-Lopez S Eckardt D Cook A Kemler R Smith A Inhibition of glycogen synthase kinase-3 alleviates Tcf3 repression of the pluripotency network and increases embryonic stem cell resistance to differentiation Nat Cell Biol 2011 13 838 845 10.1038/ncb2267 21685889 PMC3160487

[B32] Ying QL Wray J Nichols J Batlle-Morera L Doble B Woodgett J Cohen P Smith A The ground state of embryonic stem cell self-renewal Nature 2008 453 519 523 10.1038/nature06968 18497825 PMC5328678

[B33] Kiyonari H Kaneko M Abe S Aizawa S Three inhibitors of FGF receptor, ERK, and GSK3 establishes germline-competent embryonic stem cells of C57BL/6 N mouse strain with high efficiency and stability Genesis 2010 48 317 327 20162675 10.1002/dvg.20614

